# Sex and Age Differentially Regulate the Gut Microbiota and Gut Homeostasis in Mice

**DOI:** 10.1093/geroni/igaf122.2884

**Published:** 2025-12-31

**Authors:** Megan Wong, Audrey Pierce, Kerry Wellenstein, Jennifer Lee

**Affiliations:** Tufts University School of Medicine, Medford, Massachusetts, United States; Tufts University School of Medicine, Medford, Massachusetts, United States; JM-USDA HNRCA at Tufts University, Boston, Massachusetts, United States; The JM-USDA Human Nutrition Research Center on Aging at Tufts University, Boston, Massachusetts, United States

## Abstract

The gut microbiota and gastrointestinal tract serve essentials roles in regulating host metabolism, but our understanding of how sex and age contribute to these processes remains unclear. We metabolically phenotyped young (6-month-old) and old (16-23-month-old) C57BL6 female and male chow-fed mice. We collected terminal blood for serum biochemistry and cecal contents for metabolomics. Small and large intestines were collected for histology, qRT-PCR, and immune cell levels by flow cytometry. Both sexes of old mice had higher body weight, adiposity, and plasma insulin levels versus young controls. However, old female mice had 2-fold higher adiposity versus old male mice, and this was associated with a distinct sex-specific cecal lipidome profile. Old female mice had elevated circulating levels of lipopolysaccharide and cecal short-chain fatty acids versus young female mice, and these were not different within male mice. Within old mice, levels of colonic intraepithelial TCRɤδ, a key gut barrier regulator, and CD4+ T-cell cytokines were higher in females versus male mice. By contrast, old male mice had increased expression of intestinal barrier genes versus young male mice, with fewer genes differentially regulated in female mice. Similarly, colon mucus thickness and mucus protein levels were higher in old male mice versus young male controls but not different within female mice. These data identify distinct sex- and age-specific effects on multiple components of the gut microbiome and gut homeostasis that may regulate host metabolism. These differences may lead to sex-specific gut-based strategies to improve host metabolism across the lifespan.

